# ﻿A new epigean species of *Trichopeltis* Pocock, 1894 from southwest China (Diplopoda, Polydesmida, Cryptodesmidae)

**DOI:** 10.3897/zookeys.1216.128080

**Published:** 2024-10-18

**Authors:** Zhenfei Wu, Sihang Zhang, Fuxue Qin, Peiyun Cong

**Affiliations:** 1 Yunnan Key Laboratory for Palaeobiology & MEC International Joint Laboratory for Palaeobiology and Palaeoenvironment, Institute of Palaeontology, Yunnan University, Kunming 650500, China Yunnan University Kunming China; 2 Earthquake Prevention and Disaster Reduction Bureau of Zhaoyang, Zhaotong 657099, China Earthquake Prevention and Disaster Reduction Bureau of Zhaoyang Zhaotong China

**Keywords:** Key, millipedes, taxonomy, Yunnan

## Abstract

A new species of Cryptodesmidae, *Trichopeltisjiyue***sp. nov.**, is described from the Ailaoshan National Nature Reserve in Yunnan Province, southwest China. The new species is distinguished from its congeners by the gonopodal coxae with two conspicuous wing-like processes, the relatively long, stout setae on the gonopodal coxae, gonopodal telopodites glabrous and four-branched, and the acropodite curved caudolaterad. The new species is the second record of an epigean species of genus *Trichopeltis* Pocock, 1894 in China. An updated key is provided to all 14 presently known species.

## ﻿Introduction

The Polydesmida is one of the most diverse orders of Diplopoda (millipedes), containing about 5000 species in 30 families (Brewer et al. 2012) and with many species globally widespread (Shelley 2003). All Polydesmida are blind and eyeless, and metaterga usually show small to prominent lateral paranota or paraterga (Brewer and Bond 2013).

The Cryptodesmidae Karsch, 1880 is a relatively small family of Polydesmida comprising approximately 40 genera and 130 species (Golovatch et al. 2010; Liu et al. 2017). It occupies three geographic areas: Neotropical (Mexico to Argentina), Afrotropical (continental sub-Saharan Africa), and Asian + Australasian (Central Asia and the Himalayas to Japan and Papua New Guinea) (Golovatch and VandenSpiegel 2017). In tropical or subtropical Asia and Australasia, 12 genera and 36 species have been documented in Cryptodesmidae (Liu et al. 2017). The diagnosis of Cryptodesmidae has been revised by Enghoff et al. (2015) as follows: body incapable of volvation, strongly flattened; collum strongly enlarged, flabellate, with radiating lines; paraterga strongly developed, broad and subhorizontal; pore formula normal, but deviating; ozopores absent, or present on small tubercles, removed from lateral edge of paraterga; metaterga without cerotegument, densely setose and/or uniformly tuberculate, arranged in numerous transverse rows; limbus microcrenulate; epiproct exposed, from rather simple and subconical to strongly flattened and deeply incised at lateral edges; legs without sphaerotrichomes; and gonopods without seminal chamber, often with a hairy pulvillus (Enghoff et al. 2015).

*Trichopeltis* Pocock, 1894 is one of the tropical or subtropical genera of Asian Cryptodesmidae. Currently, this genus encompasses 13 species, mainly documented in Indonesia, Myanmar, Laos, Vietnam, Cambodia, southern China, and the Himalayas (Golovatch 2015, 2016; Golovatch and VandenSpiegel 2017; Likhitrakarn et al. 2017; Liu et al. 2017; Liu and Wynne 2019). This genus is well defined and characterized by a tripartite or deeply notched gonopod telopodite, including a small middle to caudal solenomere branch (Golovatch et al. 2010). Six species of this genus have been reported from China, including five cavernicolous and one epigean species.

In this paper, we describe a new epigean species of *Trichopeltis* from southwest China and update the key to all known species in this genus. This new species represents the second record of an epigean species of *Trichopeltis* in China.

## ﻿Materials and methods

All specimens were collected from the Ailaoshan National Nature Reserve (24°32'N, 101°01'E, 2476 m above mean sea level) in Yunnan Province, southwest China. Yunnan Province is well known for its high biodiversity (Yang et al. 2004). Ailaoshan Mountain National Nature Reserve stretches across six counties, or cities, of Yunnan, and is mainly covered with mid-montane humid evergreen broad-leaved forest with abundant wild fauna and flora resources (Qiu et al. 1998). The subtropical evergreen broadleaved forest is old-growth (>300 years) and well protected (Yang et al. 2007). All collected millipedes are preserved in 75% ethanol. The holotype and paratypes are deposited in Yunnan University, China.

The live photographs were taken in the habitats of the described species using a SONY DSC-RX1R camera. All specimens were further studied and photographed with a Nikon SMZ25 stereomicroscope and Nikon DS-Ri2 microscope camera within the laboratory. Scanning electron microscope (SEM) images were taken with a FEI Quanta FEG 650 with gold coating. All figures are prepared with Affinity Photo v. 2 and Affinity Designer v. 2. The terminology used here follows that of Golovatch and VandenSpiegel (2017).

## ﻿Results

### ﻿Taxonomy

**Order Polydesmida Leach, 1815**

**Family Cryptodesmidae Karsch, 1880**

**Genus *Trichopeltis* Pocock, 1894**

#### ﻿ Trichopeltis jiyue sp. nov.

Taxon classificationAnimaliaPolydesmidaCryptodesmidae

55752B89-A045-5C0D-B377-7486D658CA67

https://zoobank.org/77367C20-B420-457F-A095-AB493A6BB2F7

##### Type material.

***Holotype***: • ♂ (YNU-MD 0151), China, Yunnan Province, Pu’er City, Jingdong Yi Autonomous County, 24°54'78"N, 101°03'58"E, 2450 m elev., 4.X.2021, leg. Peiyun Cong, Sihang Zhang, Zhenfei Wu & Fuxue Qin. ***Paratypes***: • 4 ♂, 9 ♀ (YNU-MD 0152-165) same location as the holotype.

##### Etymology.

Jiyue (Chinese spelling) alludes to the bright white appearance when the animal emerges from the leaf mold, like the moon appearing from behind a dark rain cloud.

##### Diagnosis.

*Trichopeltis* is characterized by the relatively long and stout setae on the gonopodal coxae, with the posterior part having two conspicuous wing-like processes (cxp); gonopodal telopodites glabrous and four-branched; and the acropodite curved caudolaterad. The living animal is uniformly bright white.

##### Description.

Length of ♂ ca 17.2–17.8 mm, paratype ♀ ca 17.0–17.4 mm, width of midbody pro- and metazonae 2.2–2.4 mm and 5.3–5.4 mm (♂), 2.2–2.5 mm and 5.1–5.4 mm (♀), respectively.

**Figure 1.**
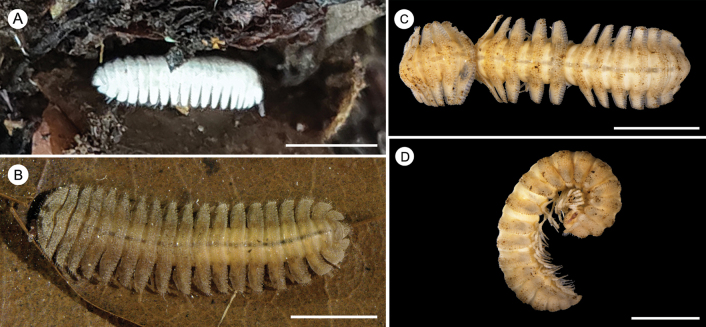
External morphology and colouration of *Trichopeltisjiyue* sp. nov. **A** ♂ holotype in habitus and live **B** fed 1–2 months in laboratory **C, D** ♀ paratype, after 3 months storage in 75% alcohol. Scale bars: 10 mm (**A**); 5 mm (**B–D**).

***Coloration*** of tergites uniformly bright white (Fig. 1A); fed 1–2 months with local mor and leaves, yellow (Fig. 1B); in alcohol, after months of preservation, whitish-yellow to yellow (Fig. 1C, D). Antenna whitish-yellow (proximal) to reddish-purple (distal).

***Adults body*** with 20 segments, collum plus 17 podous and 1 apodous tergites, plus 1 telson. In width, head << collum < segment 2 < 3 < 4 < 5 < 6 < 7-17, thereafter body tapered towards telson.

**Figure 2.**
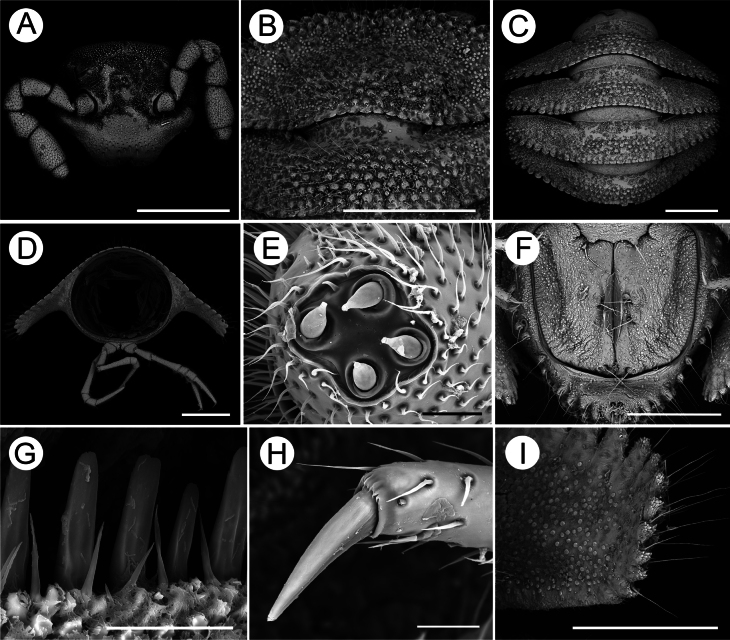
SEM images of *Trichopeltisjiyue* sp. nov., ♂ holotype **A** head, dorsal view **B** collum and the second segment, dorsal view **C** segments 6–9, dorsal view **D** cross-section of segment 5, caudal view **E** antenna disc coeloconic sensilla, plan view **F** telson and anal, ventral view **G** limbus of segment 5, subventral view **H** claw of leg, subventral view **I** paraterga of segment 6, ventral view. Scale bars: 1 mm (**A–D**); 50 μm (**E, H**); 20 μm (**G**); 500 μm (**F, I**).

***Head*** sparsely pilose, epicranial suture present (Fig. 2A). Antennae short and clavate, reaching tergite 4 when stretched ventrally; in length, antennomere 6 > 3 > 2 = 4 > 5 > 1 > 7 (Fig. 2A); antennomeres 5–7 each with a bacilliform sensilla field apico-laterally, the numbers of bacilliform sensilla are 100, 67, and 34, respectively.

***Collum*** completely covering the head from above, inverted subtrapeziform, regularly convex at peripheric margin, caudal margin slightly concave (Fig. 2B); arranged with 12 or 13 regular, transverse rows of small, spherical, setigerous tubercles on the surface, tubercles 8-13+8-13 per row, surrounded with spherical granulations, seta on each tubercle directed caudad (Fig. 2B).

***Prozona*** of segments following collum finely shagreened, metazona densely tuberculate and setose; fore and caudolateral margins of collum, anterolateral, lateral and caudal margins of following paraterga of segments besides telson with obvious dentiform-lobulate lobules, smallest at mid-dorsal region and slightly larger bidirectionally at caudal margins of paraterga.

***Dorsum*** convex, postcollum paraterga flat, very broad and long, narrowly rounded laterally, axial line absent. Metatergal segments 2–16 with four or five irregular transverse rows of similarly small, spherical, setigerous tubercles. Tubercles decreasingly extend to paraterga, but each of the latter only with three or four irregular rows of similar tubercles (Fig. 2C), surrounded by spherical granulations, same to collum; following metatergal segments 17 and 18 with 6–8 rows of smaller tubercles.

***Paraterga*** very strongly developed (Fig. 2C), regularly declivous, the tips extending down below level of venter (Fig. 2D). Segments 2–15 slightly projecting forward, each with 6–9 small, crown-like dentiform, lateral lobules (Fig. 2I) and 7–9 tongue-shaped to squarish caudolateral lobules; all evident, setigerous and microvillose segments 16–19 projecting caudally, each with 5–7 small, crown-like dentiform, lateral lobules and 9–13 tongue-shaped to squarish, caudolateral lobules; all evident, setigerous, and microvillose.

***Sterna*** sparsely setose; axial line present; tergite stricture divided into pro- and metazone parts. Limbus, with a row of tongue-shaped lobules, microdenticulate apically (Fig. 2G). Pore visible, lying on the ventral paraterga of segment 5, ozopores formula not discernable.

***Telson*** (Fig. 2F) conical, with numerous spherical granulations; epiproct flattened dorsoventrally, microtuberculate, with four strong apical papillae. Hypoproct roundly subtrapeziform, 1+1 caudal setae separated, surface rugged.

***Legs*** (Fig. 2D, H) long and slender, without modifications, longer than paraterga when stretched straight, about 1.2 times as long as the width of paraterga. In length, femur ≈ tarsus >> prefemur > coxa = tibia > postfemur > claw.

**Figure 3.**
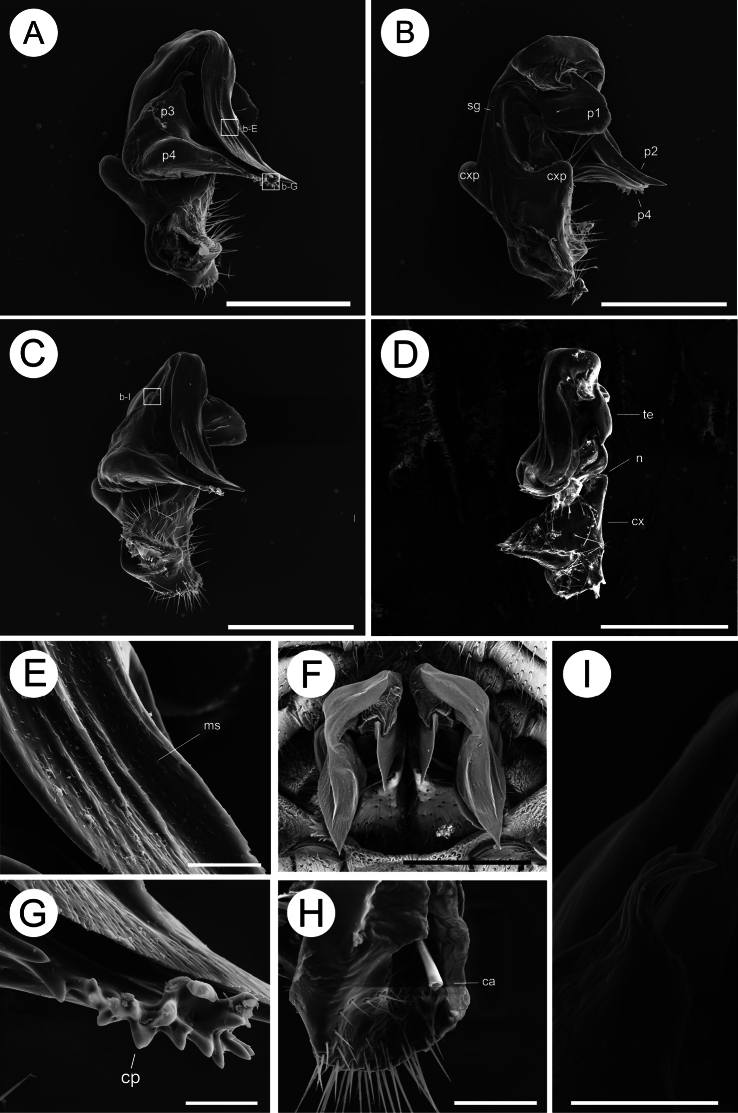
Gonopodal characters of *Trichopeltisjiyue* sp. nov., ♂ holotype, paratype. **A, C** right gonopod, sublateral and subfrontal views **B** left gonopod, subcaudal view **D** right gonopod, ventral view, coxite and telopodite divided by a notch **E** enlargement of box-E of A, prefemur sheet micro-setose on the surface **F** gonopod, ventral view from above **G** enlargement of box-G of A, corolliform solenomere **H** seminal groove **I** box-I of C, tripartite apical process. Abbreviations: p1, p2, p3, p4 = processes of telopodite; cp = corolliform pulvillus; cxp = coxa process; ms = microsetae; sg = seminal groove; tap = tripartite apical process; ca = cannula; cx = coxa; te = telopodite; n = notch. Scale bars: 500 μm (**A–D, F**); 50 μm (**E, G**); 100 μm (**H, I**).

**Figure 4.**
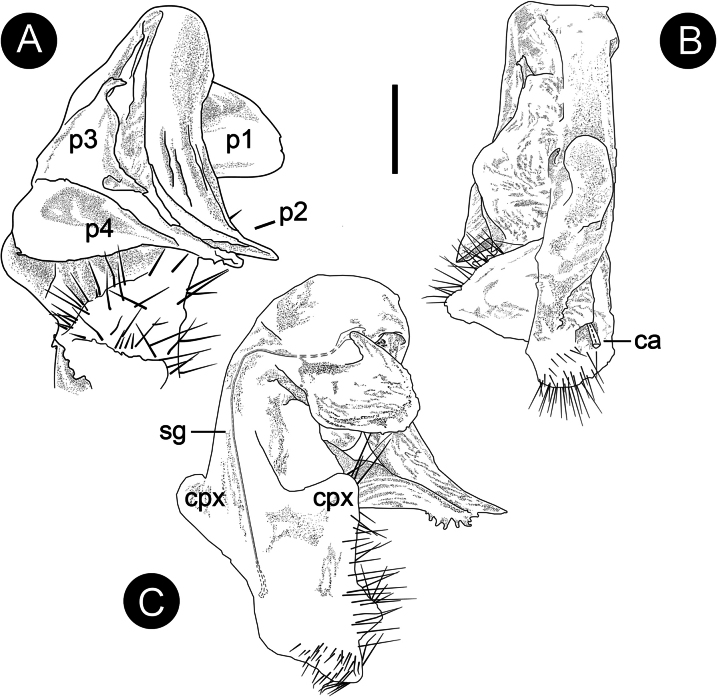
*Trichopeltisjiyue* sp. nov., ♂ holotype, paratype. **A** subfrontal view **B** subcaudal view **C** cadual view. Abbreviations: p1, p2, p3, p4 = processes of telopodite; cxp = coxa process; sg = seminal groove; ca = cannula. Scale bar: 200 μm.

***Gonopods*** complex (Figs 3, 4). Coxae with relatively long stout setae; with two conspicuous wing-like processes (cxp). Telopodite complex, with four-branched process (**p**), clearly curved (Figs 3, 4), approximately as long as coxa, divided by a notch (Fig. 3D); prefemur glabrous; femorite (**p1**) one leaf-shaped lobe on the inner side mesally; branch **p2** leaf-shaped, three times as long as **p1**, rather thick, curved caudolaterally, with dense micro-setae on surface, distal margin with serrate process; **p3** subconical, with three apical processes; **p4** leaf-shaped, close to the **p2**, the distal margin consists of numerous conical processes, forming a corolliform pulvillus; with no distinct solenomere.

##### Remarks.

The specimens were found on a stoney roadside, which some researchers usually walk around. As compared with virgin forests, the surroundings were relatively densely populated. However, the environment is undeveloped, and it the new species seemed abundant.

### ﻿Key to species of *Trichopeltis*

Modified after Liu et al. 2017.

**Table:** 

1	Tegument unpigmented, pallid to light yellowish; cavernicolous species	**2**
–	Tegument clearly pigmented, bright white, red- or grey-brown to blackish; epigean species	**7**
2	Central parts of metaterga with 2–4 irregular, transverse rows of setigerous tubercles; gonopodal coxite as usual, at most with only few setae	**3**
–	Central parts of metaterga with 5–6 irregular transverse rows of setigerous tubercles; gonopodal coxite unusually densely setose on lateral side; Yunnan, China	**6**
3	Paraterga declivous; tergal setae very long, about half as long as body diameter; gonopodal telopodite clearly twisted. Guizhou, China	***T.latellai* Golovatch et al., 2010**
–	Paraterga clearly upturned; tergal setae much shorter; gonopodal telopodite untwisted	**4**
4	Gonopodal telopodite with a hairy pulvillus; coxite short and squarish, without seta; central parts of metaterga with 4 irregular, transverse rows of setigerous tubercles. Guangxi, China	***T.liangfengdong* Liu & Wynne, 2019**
–	Gonopodal telopodite without pulvillus	**5**
5	Central parts of metaterga with 2–3 subregular, transverse rows of setigerous tubercles; acropodite strongly condensed, tripartite	***T.reflexus* Liu, Golovatch & Tian, 2017**
–	Central parts of metaterga with 3–4 irregular transverse rows of setigerous tubercles; Telopodite only slightly curved caudad, vaguely tripartite. Laos	***T.cavernicola* Golovatch, 2016**
6	Tergal setae long; gonopods relatively simple	***T.bellus* Liu, Golovatch & Tian, 2017**
–	Tergal setae short; gonopods rather complex	**12**
7	Central parts of metaterga with 4–6 irregular, transverse rows of setigerous tubercles	**8**
–	Central parts of metaterga with 2–3 irregular, transverse rows of setigerous tubercles	**11**
8	Gonopodal telopodite with evident branches	**13**
–	Gonopodal telopodite without long branches	**9**
9	Central parts of metaterga with 4–5 subregular, transverse rows of setigerous tubercles; gonopodal telopodite with a conspicuous accessory seminal chamber and a pulvillus but devoid of denticles laterally or mesally. Laos	***T.muratovi* Golovatch & VandenSpiegel, 2017**
–	Central parts of metaterga with 5–6 subregular, transverse rows of setigerous tubercles; gonopodal telopodite without accessory seminal chamber but with a pulvillus, also abundantly denticulate either laterally or mesally	**10**
10	Gonopodal telopodite abundantly denticulate on lateral face. Vietnam, Laos, and Cambodia and possibly endemic to the Indochina Peninsula	***T.kometis* Attems, 1938**
–	Gonopodal telopodite abundantly denticulate on mesal face. Sumatra, Indonesia	***T.bicolor* Pocock, 1894**
11	Frontal margin of paraterga abundantly lobulated. Solenomere lobe-shaped, tip nearly pointed	***T.feae* Pocock, 1895**
–	Frontal margin of paraterga entire, not lobulated. Solenomere axe-shaped, tip pointed	***T.watsoni* Pocock, 1895**
12	Each coxa with a conspicuous, high, curved, laterally densely setose process	***T.sutchariti* Likhitrakarn et al., 2017**
–	Each coxa with a small process without setae	***T.intricatus* Liu, Golovatch & Tian, 2017**
13	Clearly 3-branched; solenomere long and slender	***T.doriae* Pocock, 1895**
–	Clearly 4-branched; with two conspicuous wing-like process (wp) basal, one pan-shaped lobe on the inner side; acropodite reverse caudally against femorite, unfolded into sheet form. Yunnan, China	***T.jiyue* sp. nov.**

### ﻿Comparisons

The Cryptodesmidae comprises about 40 genera. The new species can be assigned to the genus *Trichopeltis* Pocock, 1894 based on the lobulated and tuberculate-setose tergites, subcordiform gonopod aperture, four-branched telopodite, and coxa divided by a notch.

Amongst all known 14 species of *Trichopeltis*, *T.jiyue* sp. nov. is most similar to *T.kometis* Attems, 1938 (Golovatch & Akkari, 2016), *T.doriae* Pocock, 1895, *T.intricatus* Liu, Golovatch & Tian, 2017, *T.sutchariti*Likhitrakarn et al., 2017, and *T.muratovi* Golovatch & Vanden Spiegel, 2017.

The gonopodal telopodite of *T.jiyue* sp. nov. clearly differs from that of *T.doriae* in having four branches, in contrast to the gonopodal telopodite of *T.doriae* which bears three branches; also, it differs from *T.muratovi* in the telopodite, which has a conspicuous accessory seminal chamber, in contrast to the gonopodal telopodite of *T.jiyue* sp. nov., which is without a conspicuous accessory seminal chamber; furthermore, the gonopodal surface of the new species is relatively smooth, with dense microsetae, and differs from that of the abundantly denticulate gonopodal surface of *T.kometis* and *T.bicolor* Pocock, 1894.

*Trichopeltisintricatus* and *T.sutchariti* were also found in Yunnan Province, China. Compared to *T.jiyue* sp. nov., the body size of *T.jiyue* sp. nov. is much larger; the length of the adult is over 17 mm, the pro- and metazonae are over 2 and 5 mm long, respectively, which is much longer than *T.intricatus*. *T.intricatus* is relatively short, ca 10 mm long, with the width of midbody pro- and metazonae 1.5 and 2.5 mm, respectively. Furthermore, the tuberculations on the collum have up to 12 or 13 irregular, transverse rows, which is more differ than the eight to nine irregular, transverse rows of *T.sutchariti*. The characters of the gonopod reveal many obvious interspecific differences.

## ﻿Conclusions

*Trichopeltisjiyue* sp. nov. is described from Ailaoshan National Nature Reserve in Yunnan Province, southwest China. It represents the second record of an epigean species of the genus *Trichopeltis* in China. An updated identification key (modified from Liu et al. 2017) to all known species of *Trichopeltis* is provided here.

## Supplementary Material

XML Treatment for Trichopeltis jiyue

## ﻿Additional information

### Author ORCIDs

Zhenfei Wu https://orcid.org/0009-0004-5586-6511

Sihang Zhang https://orcid.org/0000-0002-8230-6892

Fuxue Qin https://orcid.org/0009-0001-1140-3927

Peiyun Cong https://orcid.org/0000-0001-9910-6478

### Data availability

All of the data that support the findings of this study are available in the main text.
